# Creatinine clearance/eGFR ratio: a simple index for muscle mass related to mortality in ICU patients

**DOI:** 10.1186/s12882-024-03760-2

**Published:** 2024-10-02

**Authors:** Meint Volbeda, Hendrik W. Zijlstra, Adrian Post, Jenny E. Kootstra-Ros, Peter H. J. van der Voort, Casper F. M. Franssen, Maarten W. Nijsten

**Affiliations:** 1 Department of Critical Care, University of Groningen, University Medical Center Groningen, P.O. Box 30.001, EB70, 9700 RB Groningen, The Netherlands; 2 Department of Nephrology, University of Groningen, University Medical Center, Groningen, The Netherlands; 3 Department of Laboratory Medicine, University of Groningen, University Medical Center, Groningen, The Netherlands

**Keywords:** Estimated glomerular filtration rate, Creatinine clearance, Critically ill patients, Muscle mass, Sarcopenia, Urinary creatinine excretion, Mortality

## Abstract

**Introduction:**

In patients admitted to the intensive care unit (ICU), muscle mass is inversely associated with mortality. Although muscle mass can be estimated with 24-h urinary creatinine excretion (UCE), its use for risk prediction in individual patients is limited because age-, sex-, weight- and length-specific reference values for UCE are lacking. The ratio between measured creatinine clearance (mCC) and estimated glomerular filtration rate (eGFR) might circumvent this constraint. The main goal was to assess the association of the mCC/eGFR ratio in ICU patients with all-cause hospital and long-term mortality.

**Methods:**

The mCC/eGFR ratio was determined in patients admitted to our ICU between 2005 and 2021 with KDIGO acute kidney injury (AKI) stage 0–2 and an ICU stay ≥ 24 h. mCC was calculated from UCE and plasma creatinine and indexed to 1.73 m^2^. mCC/eGFR was analyzed by categorizing patients in mCC/eGFR quartiles and as continuous variable.

**Results:**

Seven thousand five hundred nine patients (mean age 61 ± 15 years; 38% female) were included. In-hospital mortality was 27% in the lowest mCC/eGFR quartile compared to 11% in the highest quartile (*P* < 0.001). Five-year post-hospital discharge actuarial mortality was 37% in the lowest mCC/eGFR quartile compared to 19% in the highest quartile (*P* < 0.001). mCC/eGFR ratio as continuous variable was independently associated with in-hospital mortality in multivariable logistic regression (odds ratio: 0.578 (95% CI: 0.465—0.719); *P* < 0.001). mCC/eGFR ratio as continuous variable was also significantly associated with 5-year post-hospital discharge mortality in Cox regression (hazard ratio: 0.27 (95% CI: 0.22—0.32); *P* < 0.001).

**Conclusions:**

The mCC/eGFR ratio is associated with both in-hospital and long-term mortality and may be an easily available index of muscle mass in ICU patients.

**Supplementary Information:**

The online version contains supplementary material available at 10.1186/s12882-024-03760-2.

## Introduction

Muscle mass is inversely associated with mortality in several patient categories, including patients treated in the intensive care unit (ICU) [1–5]. A practical method for estimating whole-body muscle mass is urinary creatinine excretion (UCE) [6, 7]. In an earlier study we used UCE derived from 24-h urine collections in ICU patients as an estimate of muscle mass and showed that a lower UCE is strongly associated with increased mortality [8]. However, it is difficult to define normal UCE values in individual patients since specific reference values for UCE with respect to age, sex, weight and height are lacking. This constraint can likely largely be overcome by using the ratio between the measured creatinine clearance (mCC) (calculated from 24-h UCE and plasma creatinine and indexed for a body surface area (BSA) of 1.73 m^2^) and eGFR based on the CKD-EPI 2021 formula. This so-called mCC/eGFR ratio can hypothetically be viewed as an index of muscle mass. Patients with normal kidney function and a lower muscle mass compared to the average muscle mass of age- and sex-matched individuals will in general have a lower mCC/eGFR ratio. Sequential measurements of the mCC/eGFR-ratio may also serve as a tool to monitor the course of muscle mass over time. This is because the mCC is not affected by muscle loss whereas the creatinine-based eGFR progressively overestimates the true GFR in patients with ongoing muscle wasting [9]. In a recent non-ICU study in patients with chronic kidney disease (CKD) the mCC/eGFR ratio was related to mortality [10]. The prognostic relevance of the mCC/eGFR ratio (and its course during the ICU stay) has not yet been studied in ICU patients.

The first goal of this study was to investigate the association of the mCC/eGFR ratio, as a possible index of muscle mass at ICU admission, with both all-cause in-hospital and 5 year post-hospital mortality in patients with KDIGO AKI stage 0–2 (thus excluding stage 3). An additional goal was to examine the course of the mCC/eGFR ratio, UCE, mCC and eGFR during the first 30 days of ICU stay in a subgroup of patients with an ICU stay of at least 30 days.

## Materials and methods

In this observational cohort study, we included all patients admitted ≥ 24 h to the ICU of our institution between September 2005 and June 2021. When patients had repeated ICU-admissions, data was related to the time of the first ICU-admission of the last hospital admission. The mCC is more sensitive to acute changes in renal function than eGFR, i.e. the relative change of mCC is usually greater than that of eGFR [11]. Therefore and because renal replacement therapy interferes with UCE measurements, we excluded patients with acute kidney injury (KDIGO-AKI) stage 3 [12] at any time during hospital stay to avoid an importantly disturbed correlation between mCC and eGFR. Reason for ICU admission, demographics, ICU and hospital length of stay and hospital survival, as well as long-term survival were recorded. Additionally, the Acute Physiology and Chronic Health Evaluation score 4 (APACHE-IV) was recorded in a standardized and validated way using the data dictionary of the Dutch National Intensive Care Registry (NICE) [13].

We routinely collect daily 24 h urine samples in all ICU patients and measure plasma creatinine daily at 6.00 AM. From these data mCC was calculated. These laboratory results were included in this study starting from the first complete calendar day of the ICU stay. CKD-EPI 2021 eGFR was calculated according to the recent National Kidney Foundation Laboratory Engagement Working Group Recommendations for Implementing the CKD-EPI 2021 Race-Free Equations for Estimated Glomerular Filtration Rate [14]. Since the CKD-EPI 2021 eGFR equation is normalized to a BSA of 1.73 m^2^, mCC was also normalised to 1.73 m^2^. Normalizing GFR to BSA should reduce variability in GFR among individuals with normal kidney function, since kidney size is proportional to body size [15].

Baseline plasma creatinine was based on the median plasma creatinine value during 7 days prior to ICU admission and was not available in 40 patients. For the association between mCC/eGFR ratio and all-cause mortality we used the median of the laboratory results up to the first three complete calendar days of the ICU stay (depending on the available laboratory samples). For the assessment of the time courses of mCC, eGFR and mCC/eGFR ratio we used the results from the first through the 30th complete calendar day in patients with an ICU stay of at least 30 days. For patients who were discharged alive we obtained long-term post-hospital discharge survival data up to 5 years from the hospital database and, when available, from the personal records database maintained by the municipalities in the Netherlands. For clarification a list of specific variables and definitions used throughout the paper is included in the supplementary material (Table S1).

### Sensitivity analyses

To investigate the robustness of our data, we performed three sensitivity analyses. First, to assess possible bias in the mCC/eGFR ratio caused by overestimation of mCC due to increased tubular creatinine excretion in chronic kidney disease (CKD) and a greater change in mCC compared with the change in eGFR during acute changes in renal function [11] we analyzed the relation between mCC/eGFR ratio and both in-hospital and 5 year post-hospital mortality in the subgroup of patients with a baseline plasma creatinine < 110 µmol/l and who did not develop KDIGO AKI [12]. Second, to avoid possible bias in eGFR caused by extremes in BSA we performed an additional sensitivity analysis of the association between the mCC/eGFR ratio and short- and long-term mortality without correcting the mCC to a BSA of 1.73 m^2^. Third, since the CKD-EPI 2009 eGFR without race correction performs better in the European population compared to the CKD-EPI 2021 eGFR [16], we performed also a sensitivity analysis in which the CKD-EPI 2009 eGFR without race correction was used in the mCC/eGFR ratio.

### Statistical analysis

Data were analyzed using IBM SPSS Statistics for Windows version 28 (Armonk, NY, USA) and figures were created with R version 4.1.1 (Vienna, Austria) (http://cran.r-project.org/). Patient characteristics were expressed according to the mCC/eGFR quartiles. Normally distributed data were expressed as mean and SD, and skewed data expressed as medians and interquartile range (IQR). Categorical variables were compared by the chi-square test. Normally distributed continuous variables from mCC/eGFR ratio quartiles were compared by one-way ANOVA in case of a normal distribution and by Kruskal test in case of a skewed distribution. The course of mCC, eGFR, mCC/eGFR ratio and UCE in the subset of patients with a hospital admission of at least 30 days were graphically presented by lines fitted using a locally estimated scatterplot smoothing (loess) function and a shaded area corresponding to the 95% confidence interval, calculated using a t-based approximation. To visualize the continuous association of mCC/eGFR ratio with all-cause in-hospital mortality the mCC/eGFR ratio was plotted as a continuous variable against the odds of in-hospital mortality. Additionally, to visualize the continuous association of mCC/eGFR ratio with all-cause 5 year post-hospital mortality, mCC/eGFR ratio, as continuous variable, was plotted against the hazard ratio of 5 years post-hospital mortality. In a multivariable logistic regression model the association between mCC/eGFR ratio and in-hospital mortality was assessed with correction for variables that were predictors for in-hospital mortality in univariable logistic regression models. The relationship between mCC/eGFR ratio quartiles and both in-hospital mortality and 5-year post-hospital discharge mortality was examined with Kaplan–Meier survival analysis.

## Results

### Patient characteristics and outcome

Out of total of 46,652 patients admitted to our ICU, 7509 patients were included in the analysis (Figure S1). The mean age of the included patients was 61 years and 38% were female (Table 1). Median plasma creatinine prior to ICU admission was 80 µmol/l and median plasma creatinine at ICU admission was 73 µmol/l. During ICU admission incidence rates of KDIGO AKI were 20 for stage 1 and 5 for stage 2. Median (IQR) hospital stay prior to ICU-admission was 1 (0–2) days, with a median duration of ICU stay of 4 (2–8) days and median hospital stay of 17 (11–29) days. ICU-readmission occurred in 16% of patients.

**Table 1:** Baseline characteristics for all patients and per mCC/eGFR quartile

mCC/eGFR ratio	All patients	First (lowest) quartile	Second quartile	Third quartile	Fourth (highest) quartile	*P*-value
		(≤ 0.79)	(0.80 -1.02)	(1.03—1.25)	(≥ 1.26)	
Number of patients	7509	1878	1876	1878	1877	
Female (%)	38	42	39	38	32	< 0.001
Age (years) (mean ± SD)	61 (15)	63 (15)	63 (15)	61 (15)	57 (15)	< 0.001
**Reason for ICU admission**^**a**^						
Medical	7%	5%	5%	8%	11%	< 0.001
Vascular, abdominal, miscellaneous surgery	25%	26%	23%	24%	28%	
Neurosurgery	5%	3%	2%	4%	9%	
Transplant	5%	6%	7%	3%	2%	
Cardiothoracic surgery	28%	25%	34%	32%	23%	
Trauma	8%	3%	7%	8%	15%	
Miscellaneous	22%	33%	23%	21%	13%	
APACHE-IV^b^ (median (IQR))	60 (46–78)	68 (53–90)	62 (49–79)	58 (45–74)	53 (40–69)	< 0.001
ICU LOS (days) (median (IQR))	4 (2 -8)	3 (2 -8)	4 (2 -8)	4 (2 -8)	4 (2 -8)	0.66
HOS LOS (days) (median (IQR))	17 (11–29)	20 (10–37)	18 (12–29)	16 (10–28)	16 (10–25)	< 0.001
Length (m) (mean ± SD)	1.75 (0.1)	1.73 (0.1)	1.74 (0.1)	1.75 (0.1)	1.77 (0.1)	< 0.001
Weight (kg) (mean ± SD)	82 (17)	79 (18)	81 (17)	82 (17)	85 (17)	< 0.001
BMI (kg/m^2^) (mean ± SD)	26.7 (5.3)	26.3 (5.5)	26.5 (5.1)	26.7 (5)	27.2 (5.4)	< 0.001
BSA (m^2^) (mean ± SD	1.97 (0.22)	1.92 (0.22)	1.95 (0.22)	1.98 (0.22)	2.01 (0.22)	< 0.001
Baseline plasma creatinine^c^ (µmol/l) (median (IQR))	80 (63–103)	87 (66–116)	81 (66–103)	78 (63–100)	75 (61–95)	< 0.001
Plasma creatinine (µmol/L) (median (IQR))	73 (57–102)	87 (64–133)	75 (59–102)	71 (55–94)	64 (53–85)	< 0.001
CKD-EPI 2021 eGFR (ml/min/1.73 m^2^) (median (IQR))	94 (64–107)	76 (45–99)	90 (63–103)	96 (71–107)	102 (86–112)	< 0.001
mCC (ml/min/1.73 m^2^) (median (IQR))	85 (51–121)	41 (25–61)	79 (56–94)	106 (78–122)	142 (114–167)	< 0.001
UCE (mmol/24 h) (median (IQR))	10.0 (7.1–13.5)	5.7 (4.4–7.2)	9.2 (7.5–11.0)	11.8 (9.5–13.9)	15.2 (12.2–18.5)	< 0.001
Females (median (IQR))	7.8 (5.8–9.9)	4.8 (3.7–5.8)	7.4 (6.4–8.4)	9.0 (7.8–10.3)	11.2 (9.5–13.0)	< 0.001
Males (median (IQR))	11.9 (8.7–15.3)	6.8 (5.3–8.0)	10.4 (9.1–11.7)	13.3 (11.8–15.0)	17.1 (14.8–19.6)	< 0.001
Max KDIGO AKI stage						
Stage 1	20%	29%	21%	17%	12%	< 0.001
Stage 2	5%	11%	4%	3%	2%	

*mCC* measured creatinine clearance, *eGFR* estimated glomerular filtration rate, *APACHE-IV* Acute Physiology And Chronic Health Evaluation score 4, *ICU-LOS* intensive care unit length of stay, *HOS-LOS* hospital length of stay, *BMI* body mass index, *BSA* body surface area, *CKD-EPI 2021* Chronic Kidney Disease Epidemiology Collaboration (CKD-EPI) 2021 equation, *UCE* urinary creatinine excretion, *KDIGO AKI* Kidney Disease Improving Global Outcomes acute kidney injury

^a^Reason for ICU admission was missing in 4426 patients. ^b^APACHE-IV scores were missing in 606 patients. ^c^baseline plasma creatinine was based on the median plasma creatinine value during 7 days prior to ICU admission and was not available in 40 patients

Patients in lower mCC/eGFR ratio categories were older and had higher median plasma creatinine values at baseline and after ICU admission (*P* < 0.001 for all comparisons, Table 1). Median mCC and UCE increased in subsequent mCC/eGFR categories. Overall, median UCE was 34% lower in females compared to males (Table 1).

### Correlation between the mCC/eGFR ratio and UCE

**Fig. 1:**
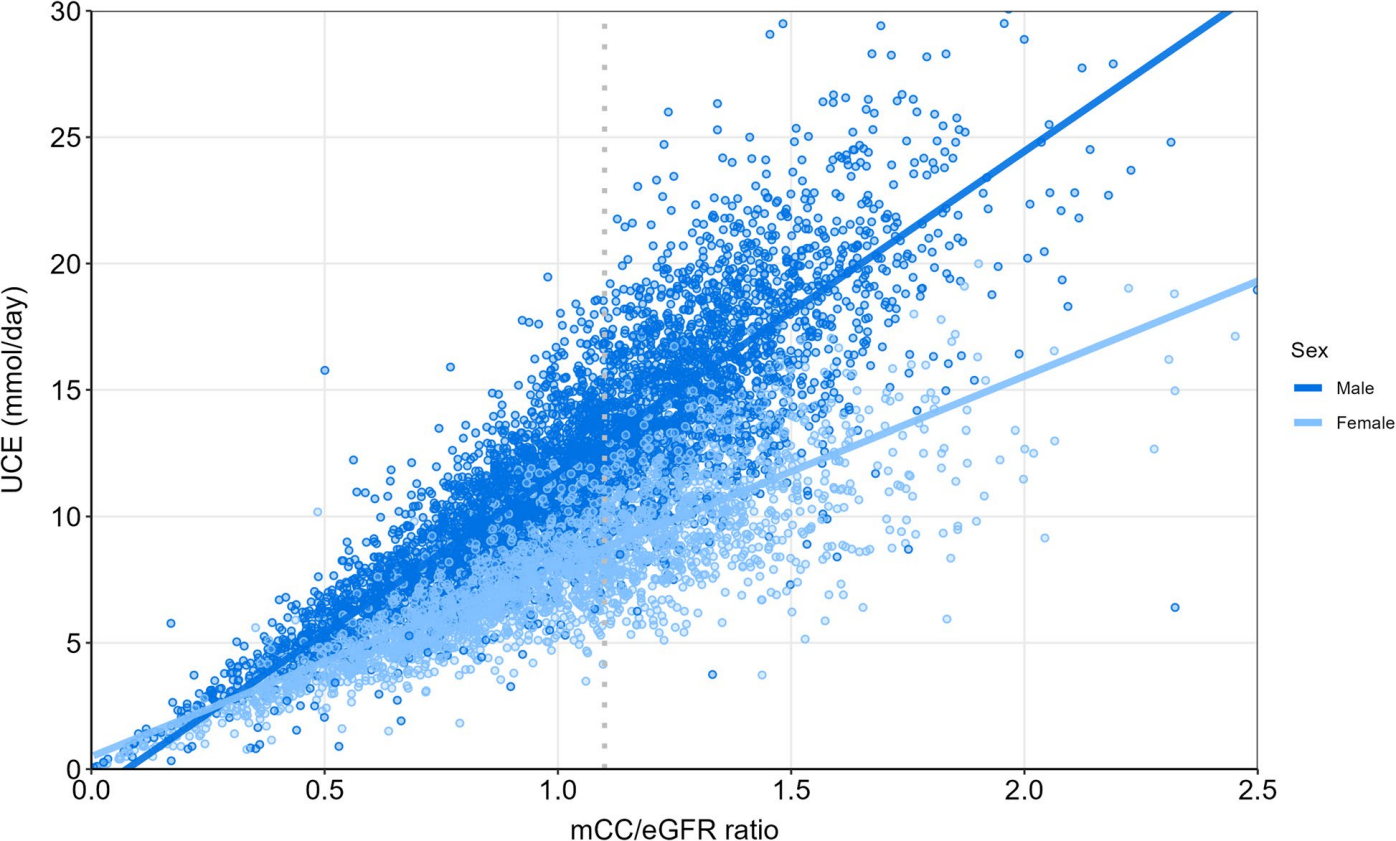
Relation between mCC/eGFR ratio and UCE in males and females. Legend: Relation between mCC/eGFR ratios and UCE at day 1 of ICU admission in males (4677, with a Pearson’s *r* = 0.88; *P* < 0.001) and females (2832, *r* = 0.84; *P* < 0.001) separately to underscore the relation between mCC/eGFR and UCE and to demonstrate the difference in UCE range between males and females at an mCC/eGFR ratio of 1.1. The different slopes between males and females are a consequence of the higher UCE in males compared to females

The mCC/eGFR ratio was strongly correlated with UCE in both males (Pearson correlation 0.88 (*P* < 0.001)) and females (Pearson correlation 0.84 (*P* < 0.001) (Fig. 1). Instead of a single UCE value corresponding to a specific mCC/eGFR ratio, there was a range of UCE values that corresponded to a specific mCC/eGFR ratio likely representing interindividual differences in age, sex, weight and height affecting UCE. The marked difference in UCE between males and females is illustrated in Fig. 1.

### Courses of mCC, eGFR and the mCC/eGFR ratio

**Fig. 2:**
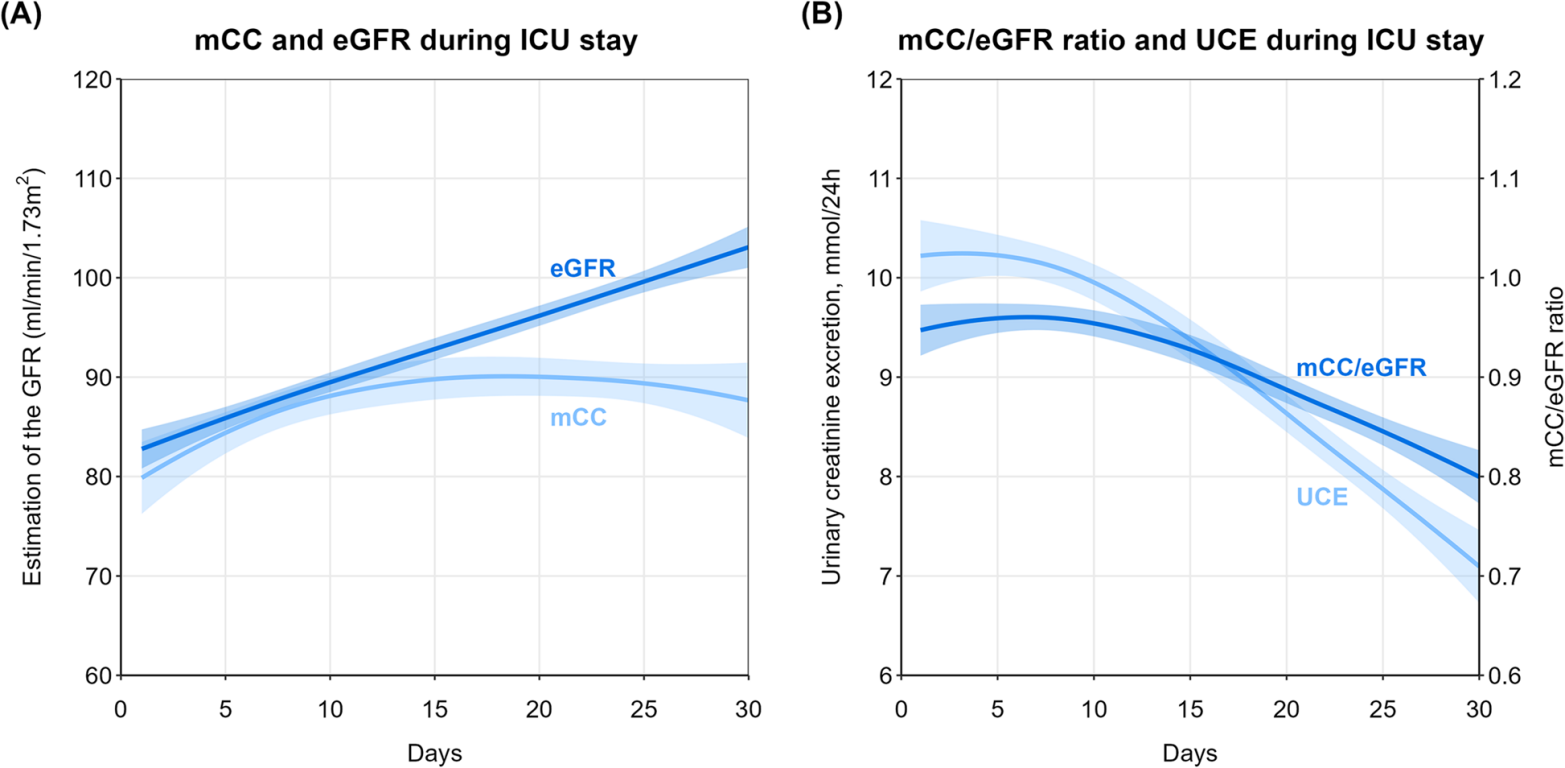
Time course of mCC, eGFR, mCC/eGFR ratio and UCE. Legend: Graphical representation of (**A**) the course of eGFR and creatinine clearance during the ICU stay and (**B**) the course of the creatinine clearance to eGFR ratio (mCC/eGFR ratio) and urinary creatinine excretion (UCE) in patients (n = 303) with an ICU stay of at least 30 days. The lines are fitted using a locally estimated scatterplot smoothing (loess) function and the shaded area corresponds to the 95% confidence interval, calculated using a t-based approximation

The courses of mCC, eGFR and mCC/eGFR ratio during the first 30 days of follow up were available in 303 patients with an ICU stay of at least 30 days. During the ICU stay, eGFR gradually increased, while mCC remained more or less stable (Fig. 2A), resulting in a progressive decline of the mCC/eGFR ratio (Fig. 2B). The decline of the mCC/eGFR ratio was accompanied by a decline in UCE, indicating loss of muscle mass (Fig. 2B).

### The mCC/eGFR ratio in relation to in-hospital and post-hospital discharge 5-year mortality

**Fig. 3:**
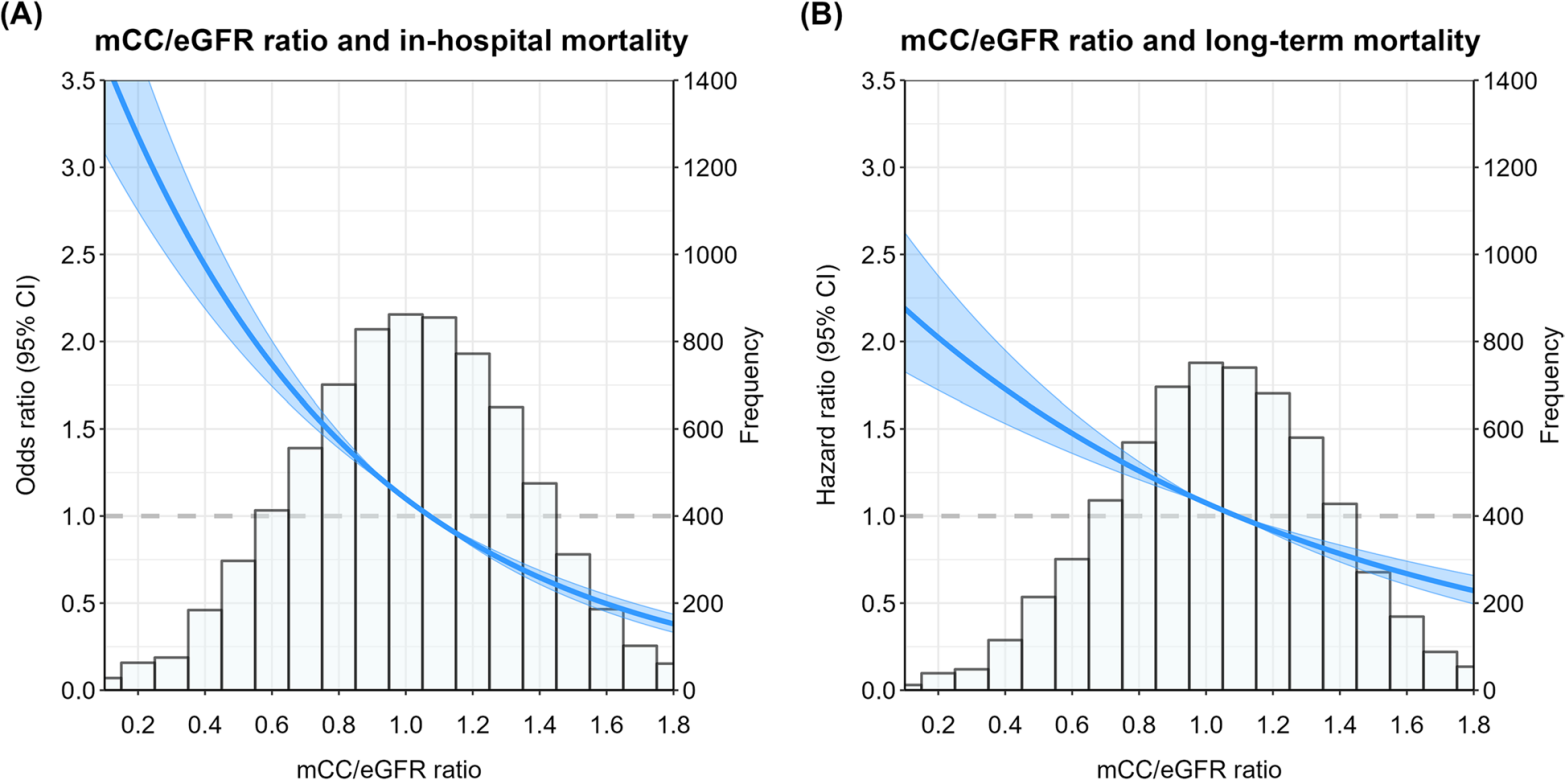
Association between the mCC/eGFR ratio and in-hospital mortality and mortality 5 year after hospital discharge. Legend: Graphical representation of the association of mCC/eGFR ratio with the risk of (**A**) in-hospital and (**B**) 5 years post-hospital discharge mortality. The lines show the odds ratio (OR) for short-term mortality and hazard ratio (HR) for long-term mortality. The shaded area corresponds to the 95% pointwise confidence interval (CI). *P*-values are < 0.001 and < 0.001 for in-hospital mortality and long-term mortality, respectively. A histogram of the mCC/eGFR ratio is plotted in the background to demonstrate the distribution of the mCC/eGFR ratio. The graph demonstrates that a lower mCC/eGFR ratio is associated with higher risk of both in-hospital and long-term mortality

Overall in-hospital mortality was 16%. In-hospital mortality was 27% in the lowest mCC/eGFR quartile, 16% in the second, 13% in the third and 11% in the highest quartile (*P* < 0.001). Lower mCC/eGFR ratios were also associated with higher odds of in-hospital mortality when the mCC/eGFR ratio was plotted as a continuous variable (*P* < 0.001) (Fig. 3A).

Overall actuarial mortality 5 year after hospital discharge was 27%. The actuarial mortality was 37% in the lowest mCC/eGFR quartile, 28% in the second, 27% in the third and 19% in the highest quartile (*P* < 0.001).

Lower mcc/eGFR ratios were associated with higher hazard ratios for 5-year post-hospital discharge mortality, when mCC/eGFR ratio was plotted as a continuous variable (*P* < 0.001) (Fig. 3B).

In a multivariable logistic regression analysis model, the mCC/eGFR ratio remained an independent predictor of in-hospital mortality (Table S2) after correction for variables that predicted in-hospital mortality in univariable logistic regression models (Table 2).

**Table 2:** Univariable logistic regression model for in-hospital mortality

Factor	B	Wald	Odds ratio Exp (B) (95% CI for exp B)	P Value
mCC/eGFR ratio	-1.326	192.946	0.266 (0.22—0.32)	< .001
UCE (mmol/24 h)	-0.100	176,991	0.905 (0.891—0.918)	< .001
CKD-EPI 2021 eGFR 2021 (ml/min/1.73 m^2^)	-0.013	166.587	0.987 (0.985—0.989)	< .001
mCC (ml/min/1.73 m^2^)	-0.01	194.297	0.99 (0.988—0.991)	< .001
Plasma creatinine (µmol/L)	0.006	121.473	1.006 (1.005—1.007)	< .001
Age (years)	0.022	97.477	1.023 (1.018—1.027)	< .001
Baseline plasma creatinine^a^ (µmol/L)	0.005	48.327	1.005 (1.003—1.006)	< .001
APACHE IV score	0.042	861.09	1.043 (1.04—1.046)	< .001
KDIGO AKI stage 1	0.737	124.77	2.089 (1.836—2.377)	< .001
KDIGO AKI stage 2	1013	79.799	2.754 (2.205—3.439)	< .001
BMI (kg/m^2^)	-0.003	0.211	0.997 (0.986—1.009)	0.646
BSA (m^2^)	-0.498	12.587	0.607 (0.461—0.8)	< .001
Length (cm)	-0.013	15.94	0.987 (0.981—0.994)	< .001
Weight (kg)	-0.004	5.967	0.996 (0.992—0.999)	0.015

*mCC* measured creatinine clearance, *eGFR* estimated glomerular filtration rate, *UCE* urinary creatinine excretion *CKD-EPI 2021* Chronic Kidney Disease Epidemiology Collaboration (CKD-EPI) 2021 equation, *APACHE-IV* Acute Physiology And Chronic Health Evaluation Score 4, *KDIGO AKI* Kidney Disease Improving Global Outcomes acute kidney injury, *BMI* body mass index, *BSA* body surface area. ^a^baseline plasma creatinine was based on the median plasma creatinine value during 7 days prior to ICU admission and was not available in 40 patients

**Fig. 4:**
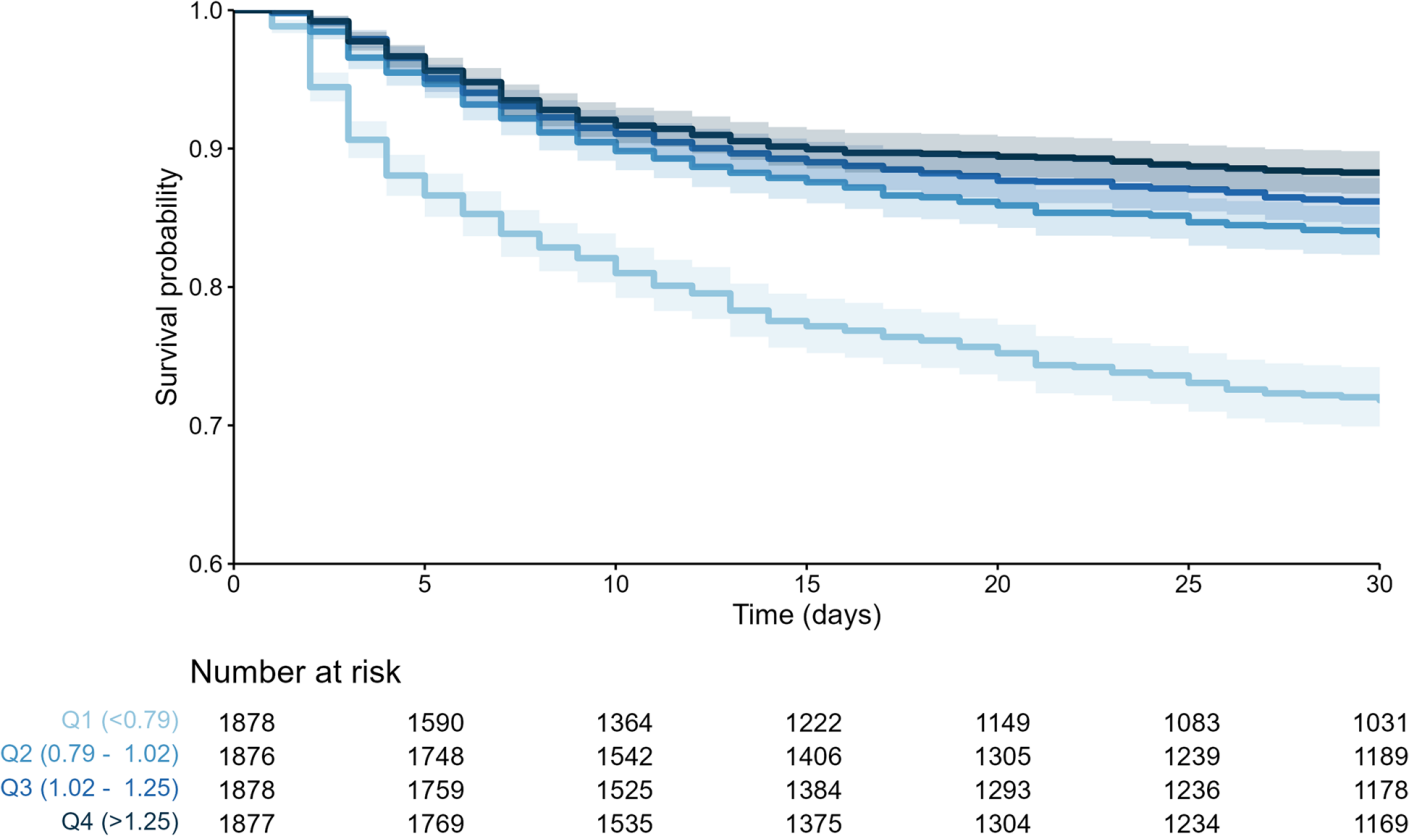
Kaplan–Meier curves over the first 30 days according to quartiles of mCC/eGFR ratio

**Fig. 5:**
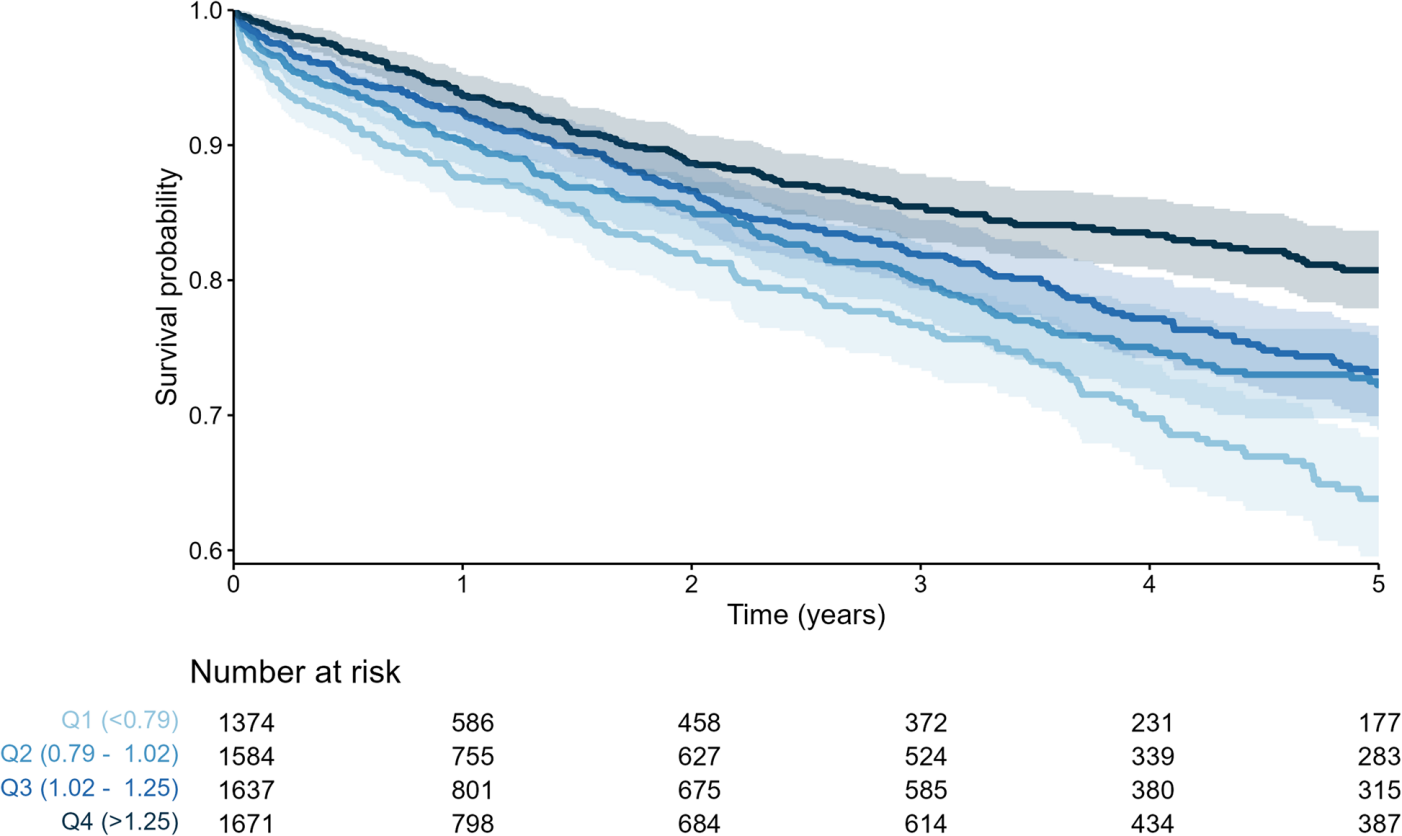
Kaplan–Meier curves of 5 year post-hospital discharge mortality according to quartiles of mCC/eGFR ratio

Kaplan–Meier analyses of the relationship between the mCC/eGFR quartiles and both in-hospital and 5-year post-hospital mortality showed significantly higher survival rates in patients with higher mCC/eGFR ratios (Fig. 4 and Fig. 5).

### Sensitivity analyses

In a sensitivity analysis in patients without KDIGO acute kidney injury and a baseline plasma creatinine < 110 µmol/l, lower mCC/eGFR ratios were persistently significantly associated with higher odds for in-hospital (*P* < 0.001) and higher hazard ratios for 5-year post-hospital discharge mortality (*P* < 0.001) (Figure S2). In an additional sensitivity analysis in which mCC was not normalised to a BSA of 1.73 m^2^, lower mCC/eGFR ratios were persistently significantly associated with both higher odds for in-hospital (*P* < 0.001) and higher hazard ratios for 5-year post-hospital discharge mortality (*P* < 0.001) (Figure S3). In a third sensitivity analysis with the CKD-EPI 2009 eGFR without race correction in the mCC/eGFR ratio, lower mCC/eGFR ratios were persistently and significantly associated with both higher odds for in-hospital (*P* < 0.001) and higher hazard ratios for mortality 5-year post-hospital discharge (*P* < 0.001) (Figure S4).

## Discussion

In this study we investigated the association between the mCC/eGFR ratio and all-cause mortality in ICU patients. The main finding is that both short-term and long-term mortality are considerably higher in patients with a lower mCC/eGFR ratio. Second there is a strong correlation between the mCC/eGFR ratio and UCE, which is an established method for assessment of whole-body muscle mass.

Univariably, the mCC/eGFR ratio was a strong predictor of in-hospital mortality. The mCC/eGFR ratio remained an independent predictor of in-hospital mortality in multivariable logistic regression analysis with correction for confounders like APACHE IV score, KDIGO AKI stage, baseline plasma creatinine, body length and weight.

The development of muscle loss during ICU stay can be detected by a decreasing mCC/eGFR ratio (Fig. 2B). As a result of gradual muscle loss during the ICU stay, plasma creatinine levels declined, resulting in increasing eGFR values, relative to the mCC levels, which are unaffected by muscle mass [9].

An mCC/eGFR ratio equal or above 1.1 could be considered representative for normal whole-body muscle mass in patients with a normal kidney function, since eGFR was derived mostly from ‘true’ iothalamate clearance whereas mCC overestimates the true glomerular filtration rate by approximately 10%, due to tubular secretion of creatinine in addition to glomerular filtration [17]. In patients with decreased kidney function tubular secretion of creatinine relatively increases [18] likely resulting in an even higher ‘cut-off value’ for the mCC/eGFR ratio. In our cohort, tubular secretion was not considered to be importantly increased in most patients, since the median baseline creatinine was 80 (IQR 64–103) µmol/l and AKI stage 3 patients were excluded. When we consider an mCC/eGFR ratio equal or above 1.1 representative for normal whole-body muscle mass, 43% of males and 63% of females in our study had a diminished whole-body muscle mass at ICU admission.

UCE is seen as an established method for estimating whole-body muscle mass since creatinine is generated in muscle cells proportional to muscle mass and is subsequently transported to the blood and eventually excreted in urine [6]. Compared to computed tomography and magnetic resonance imaging in which only some selected muscle groups are measured, UCE is a representation of the whole body muscle mass [19]. In kidney transplant patients UCE is also related to muscle performance [20]. In earlier publications, we demonstrated that lower muscle mass as reflected by lower UCE is related to in-hospital and long-term mortality [8] and that muscle-mass expressed as UCE decreases during ICU stay [9]. In non-ICU patients after liver transplantation a relationship between lower UCE and mortality was also observed [21].

Although the prevalence of sarcopenia in ICU patients is high, a gold standard for the assessment of sarcopenia in ICU patients is lacking [22]. Further studies are needed to investigate if the mCC/eGFR ratio can be used to reliably detect sarcopenia in ICU patients and whether or not the effects of interventions such as physical therapy or long-term use of neuromuscular blockage agents can result in changes in the mCC/eGFR ratio. Further research is necessary to investigate if in obese patients the mCC/eGFR ratio has the same correlation with UCE, since (A) an eGFR formula standardized to a body surface area of 1.73 m^2^ underestimates the actual GFR in obese patients and (B) in obese patients total body water involved in glomerular filtration does not increase relative to the increase in body weight. Currently mCC is not routinely measured in most ICUs. However, since plasma creatinine and eGFR formula’s overestimate renal function in ICU patients due to loss of muscle mass, adding mCC to the ICU’s laboratory diagnostic routine not only improves assessment of renal function in steady state situations, but as part of the mCC/eGFR ratio probably also improves assessment of whole-body-muscle-mass.

In this study, we normalized mCC to a BSA of 1.73 m^2^ since the CKD-EPI 2021 eGFR equation is also normalised to a BSA of 1.73 m^2^. However in the non-ICU study introducing the concept of the mCC/eGFR ratio, mCC was not corrected for BSA [10]. Notably, in a sensitivity analysis without correcting mCC for BSA, we found a similar association between the mCC/eGFR ratio and all-cause mortality (Figure S3).

It should be noted that in this study we chose to use the race-free CKD-EPI 2021 eGFR formula to avoid a possible suggestion of racism [23]. This formula results in a median increase of 3.9 (2.9–4.8) ml/min/1.73 m^2^ in eGFR compared to the CKD-EPI 2009 eGFR formula in European populations [24]. In sensitivity analyses with the CKD-EPI 2009 formula we found a similar association between mCC/eGFR ratio and all-cause mortality (Figure S4).

The strengths of our study are that the mCC/eGFR ratio was investigated in a large ICU cohort and that it can relatively easy be applied in practice. Our study also has limitations. First, this is a retrospective analysis covering a long time period. However, laboratory data were prospectively collected and 24 h urine collections are routinely performed at our ICU which minimises the risk of misinterpretation of data. Second, although we excluded patients who developed KDIGO AKI stage 3 in a part of the selected cohort still considerable changes in renal function occurred which might have influenced the mCC/eGFR ratio. However, the exclusion of all patients with AKI would make it difficult to extrapolate our findings to real world clinical practice. Third, the mCC/eGFR ratio might also be affected by administrated drugs and fluids. Fourth, we did not compare muscle mass as assessed by the mCC/eGFR ratio with an imaging technique, for example by measuring the rectus femoris cross-sectional area [25, 26].

## Conclusion

The mCC/eGFR ratio might be useful to estimate whole-body muscle mass in ICU patients independent of demographics and is independently related to both in-hospital and long-term mortality. Future research could establish the utility of the mCC/eGFR ratio.

## Supplementary Information

Supplementary Material 1: Table S1. List of variables with abbreviations and definitions. Table S2. Multivariable logistic regression model for in-hospital mortality. Figure S1. Flow chart of patient selection. Figure S2. Sensitivity analysis of mCC/eGFR ratio in patients without KDIGO acute kidney injury (AKI) and a baseline plasma creatinine < 110 µmol/l. Figure S3. Sensitivity analysis of mCC/eGFR ratio without normalizing mCC to a body surface area of 1.73 m2. Figure S4. Sensitivity analysis of mCC/eGFR ratio with CKD-EPI 2009 eGFR without race correction.

## Data Availability

The anonymized data underlying this article will be shared upon reasonable request to the corresponding author.

## Abbreviations

APACHE-IV: Acute physiology and chronic health evaluation score 4
AKI: Acute kidney injury
BSA: Body surface area
CKD: Chronic kidney disease
eGFR: Estimated glomerular filtration rate
ICU: Intensive care unit
IQR: Interquartile range
mCC: Measured creatinine clearance
NICE: (Dutch) national intensive care registry
UCE: Urinary creatinine excretion
