# Supplementary material for: Creatinine clearance/eGFR ratio: a simple index for muscle mass related to mortality in ICU patients
Source: BMC Nephrol. 2024 Oct 2;25:330. doi: 10.1186/s12882-024-03760-2 (PMC11446022; doi:10.1186/s12882-024-03760-2)
Supplement: Supplementary file 1 — Supplementary Material 1: Table S1. List of variables with abbreviations and definitions. Table S2. Multivariable logistic regression model for in-hospital mortality. Figure S1. Flow chart of patient selection. Figure S2. Sensitivity analysis of mCC/eGFR ratio in patients without KDIGO acute kidney injury (AKI) and a baseline plasma creatinine < 110 µmol/l. Figure S3. Sensitivity analysis of mCC/eGFR ratio without normalizing mCC to a body surface area of 1.73 m2. Figure S4. Sensitivity analysis of mCC/eGFR ratio with CKD-EPI 2009 eGFR without race correction. [file 12882_2024_3760_MOESM1_ESM.docx]

**Index of the Table and Figures:**

**Table S1.** List of variables with abbreviations and definitions.

**Table S2.** Multivariable logistic regression model for in-hospital mortality.

**Figure S1.** Flow chart of patient selection.

**Figure S2.** Sensitivity analysis of mCC/eGFR ratio in patients without KDIGO acute kidney injury (AKI) and a baseline plasma creatinine <110 µmol/l.

**Figure S3.** Sensitivity analysis of mCC/eGFR ratio without normalizing mCC for body surface area.

**Figure S4.** Sensitivity analysis of mCC/eGFR ratio with CKD-EPI 2009 eGFR

without race correction.

**Table S1.** List of variables with abbreviations and definitions.

| **Abbreviation** | **Variable** | **Definition** |
| --- | --- | --- |
| CKD-EPI-2021 eGFR/1.73m^2^ | Chronic Kidney Disease Epidemiology Collaboration 2021 equation eGFR/1.73m^2^ | Race free CKD-EPI formula introduced in 2021 and normalised to a body surface area of 1.73 m^2^ |
| mCC | Measured creatinine clearance | Creatinine clearance based on plasma creatinine and total amount of creatinine excretion in mmol per 24 hours |
| mCC/1.73 m^2^ | Measured creatinine clearance/1.73 m^2^ | Measured creatinine clearance normalised to a body surface area of 1.73 m^2^ |
| mCC/eGFR ratio | Measured creatinine clearance/1.73m^2^ / Chronic Kidney Disease Epidemiology Collaboration 2021 equation eGFR/1.73 m^2^ ratio | Ratio between mCC/1.73 m^2^ and CKD-EPI-2021 eGFR/1.73m^2*^ |
| UCE | Urinary creatinine excretion | Total amount of creatinine excreted in mmol per 24 hours |

**Legend to Table S1.** All variables are determined by the median value up to the first 3 complete calendar days of the ICU stay (depending on the available laboratory samples), with two exceptions; (1) Baseline plasma creatinine (prior to admission) is based on the median plasma creatinine value during 7 days prior to ICU admission, (2) Graphs in Figure 2 include also variables after the first 3 days of ICU stay. *****Except for the sensitivity analysis in Figure S3 in which the ratio between mCC and CKD-EPI-2021 eGFR/1.73m^2^ was used.

**Table S2.** Multivariable logistic regression model for in-hospital mortality.

| **Factor** | **B** | **Wald** | **Odds ratio**  **Exp (B) (95% CI for exp B)** | **P Value** |
| --- | --- | --- | --- | --- |
| mCC/eGFR ratio | -0.548 | 24.287 | 0.578 (0.465 - 0.719) | <0.001 |
| APACHE IV | 0.041 | 712.744 | 1.042 (1.039 - 1.045) | <0.001 |
| KDIGO AKI 1 | 0.207 | 5.543 | 1.23 (1.035 - 1.461) | 0.019 |
| KDIGO AKI 2 | 0.475 | 9.903 | 1.608 (1.196 - 2.162) | 0.002 |
| Baseline plasma creatinine^1^ (µmol/L) | -0.002 | 7.683 | 0.998 (0.996 - 0.999) | 0.006 |
| Length (cm) | -0.004 | 1.185 | 0.996 (0.988 - 1.003) | 0.276 |

**Legend to Table S2.** Multivariable logistic regression model for hospital mortality based on mCC/eGFR ratio corrected for independent variables which predicted mortality in the univariable logistic regression model (Table 2). No correction was performed for variables that are part of the mCC/eGFR ratio equation: age, urinary creatinine excretion per 24 hours, eGFR, mCC and plasma creatinine at day 1 of ICU stay. mCC, measured creatinine clearance; eGFR, estimated glomerular filtration rate; APACHE-IV, Acute Physiology And Chronic Health Evaluation Score 4; KDIGO AKI, Kidney Disease Improving Global Outcomes acute kidney injury. ^1^ baseline plasma creatinine is based on the median plasma creatinine value during 7 days prior to ICU admission and was not available in 40 patients.

**Figure S1.** Flow chart of patient selection.


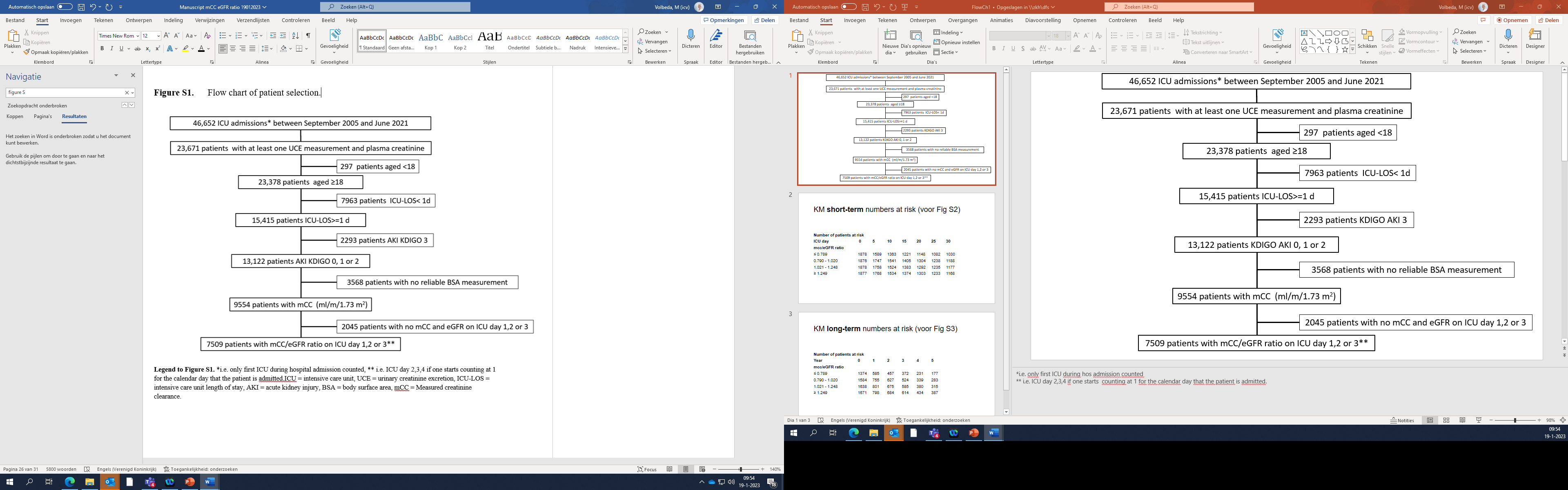


**Legend to Figure S1.** *i.e. only first ICU admission during a hospital admission was counted, ** i.e. ICU day 2,3,4 if one would start counting at 1 for the calendar day that the patient is admitted. ICU, intensive care unit; UCE, urinary creatinine excretion; ICU-LOS, intensive care unit length of stay; AKI, acute kidney injury; BSA, body surface area; mCC, measured creatinine clearance. **Figure S2.** Sensitivity analysis of mCC/eGFR ratio in patients without KDIGO acute kidney injury (AKI) and a baseline plasma creatinine <110 µmol/l.


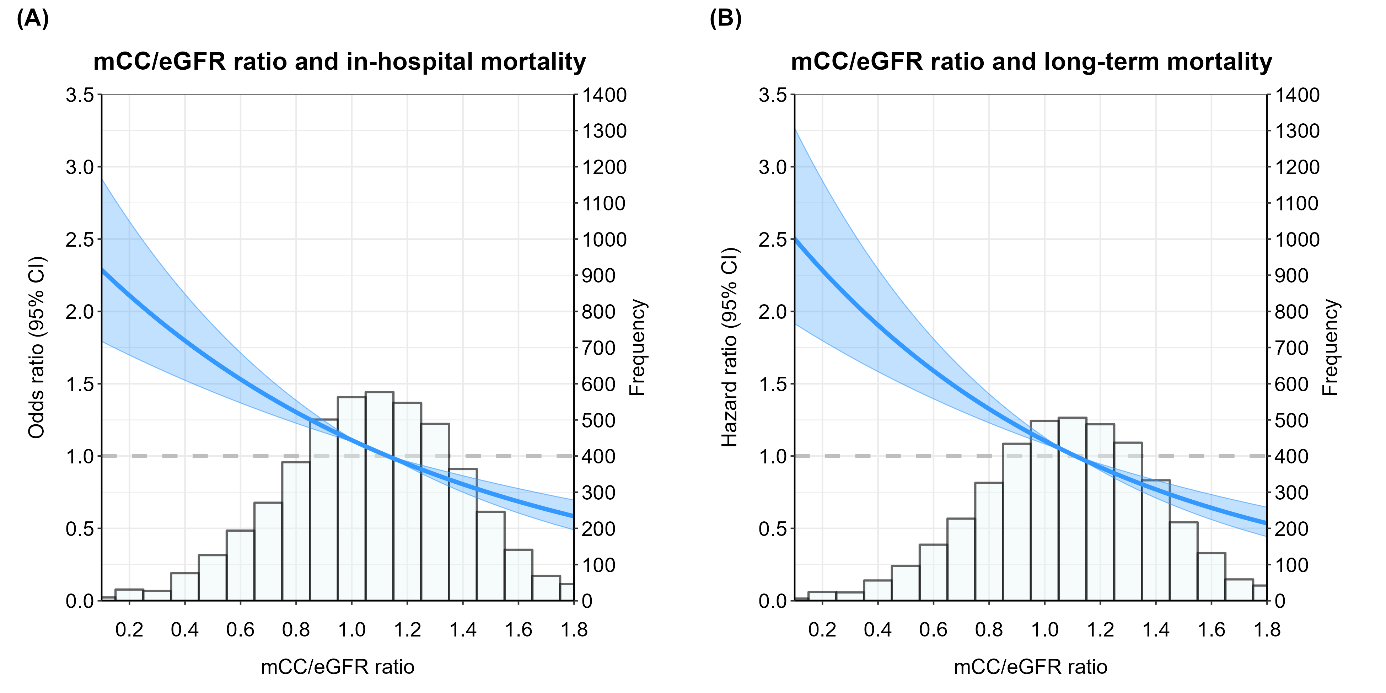


**Legend to Figure S2**. Sensitivity analyses of the association of mCC/eGFR ratio with the risk of (A) in-hospital and (B) 5 years post-hospital discharge mortality in patients without KDIGO acute kidney injury (AKI) and a baseline plasma creatinine <110 µmol/l. The lines show the odds ratio (OR) for short-term mortality and hazard ratio (HR) for long-term mortality. The shaded area corresponds to the 95% pointwise confidence interval (CI). P-effects are <0.001 and <0.001 for short- and long-term mortality, respectively. A histogram of the mCC/eGFR ratio is plotted in the background to demonstrate the distribution of the mCC/eGFR ratio.

**Figure S3.** Sensitivity analysis of mCC/eGFR ratio without normalizing mCC for body surface area.


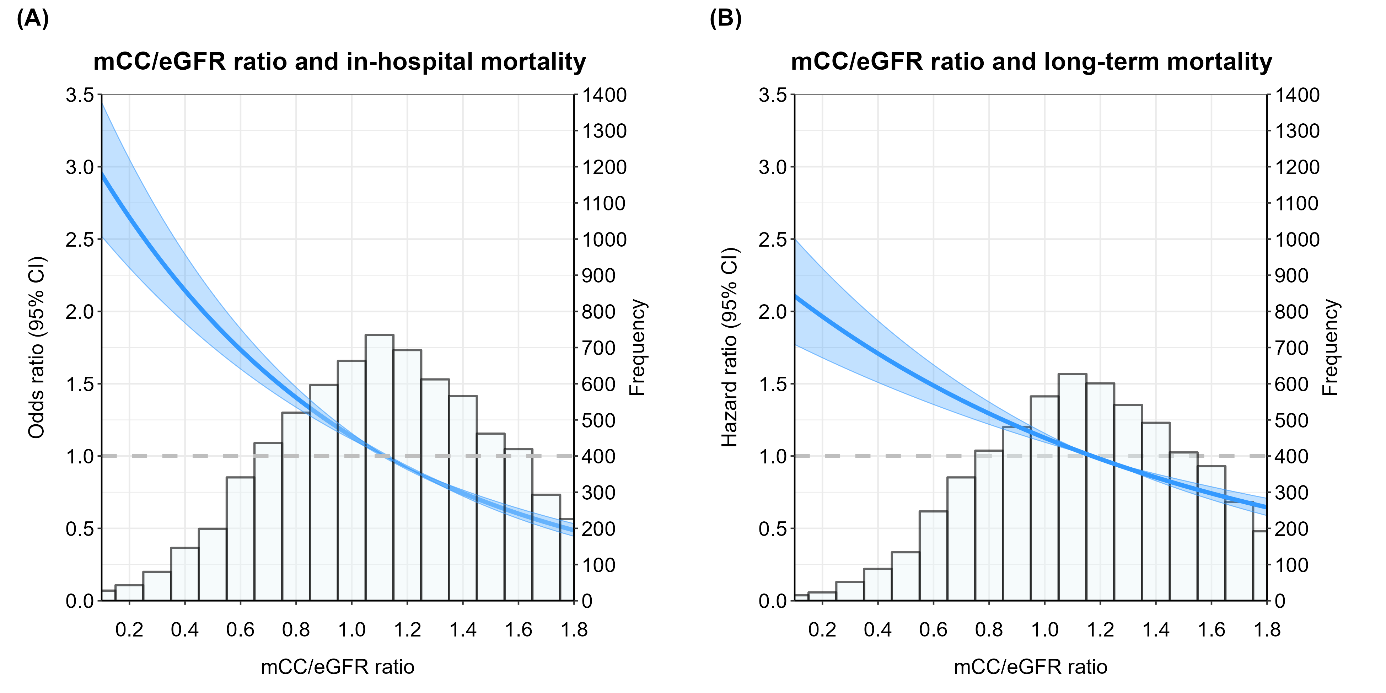


**Legend to Figure S3.** Sensitivity analyses of the association of mCC/eGFR ratio with the risk of (A) in-hospital and (B) 5 years post-hospital discharge mortality, using mCC unadjusted for BSA. The lines show the odds ratio (OR) for short-term mortality and hazard ratio (HR) for long-term mortality. The shaded area corresponds to the 95% pointwise confidence interval (CI). P-effects are <0.001 and <0.001 for in-hospital and long-term mortality, respectively. A histogram of the mCC/eGFR ratio is plotted in the background to demonstrate the distribution of the mCC/eGFR ratio.

**Figure S4.** Sensitivity analysis of mCC/eGFR ratio with CKD-EPI 2009 eGFR without race correction.


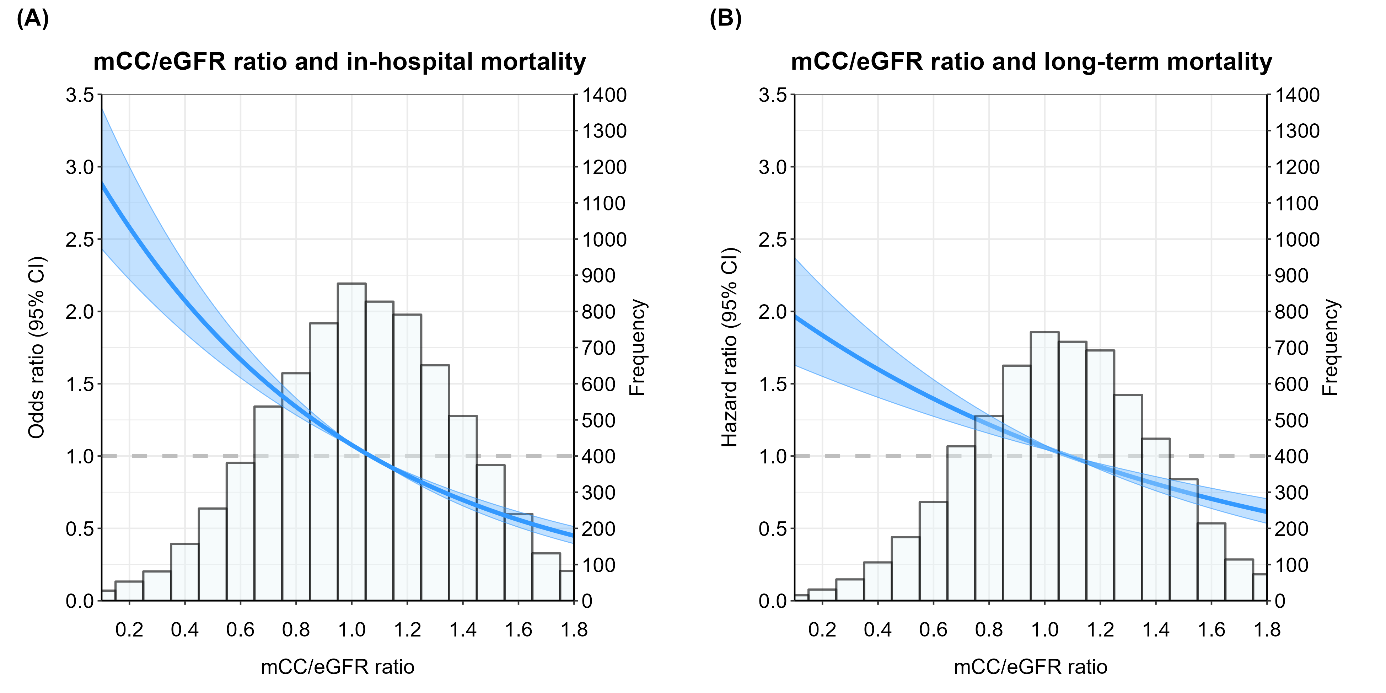


**Legend to Figure S4:** Graphical representation of the association of mCC/eGFR ratio with the risk of **(A)** in-hospital and **(B)** 5 years post-hospital discharge mortality. The lines show the odds ratio (OR) for short-term mortality and hazard ratio (HR) for long-term mortality. The shaded area corresponds to the 95% pointwise confidence interval (CI). P-effects are <0.001 and <0.001 for in-hospital mortality and long-term mortality, respectively. A histogram of the mCC/eGFR ratio is plotted in the background to demonstrate the distribution of the mCC/eGFR ratio. The graph demonstrates that a lower mCC/eGFR ratio is associated with higher risk of both in-hospital and long-term mortality.
